# Taking a machine learning approach to optimize prediction of vaccine hesitancy in high income countries

**DOI:** 10.1038/s41598-022-05915-3

**Published:** 2022-02-08

**Authors:** Tania M. Lincoln, Björn Schlier, Felix Strakeljahn, Brandon A. Gaudiano, Suzanne H. So, Jessica Kingston, Eric M.J. Morris, Lyn Ellett

**Affiliations:** 1 Clinical Psychology and Psychotherapy, Institute of Psychology, Faculty of Psychology and Movement Sciences, Universität Hamburg, Von-Melle-Park 5, 20146 Hamburg, Germany; 2 Brown University and Butler Hospital, Providence, USA; 3 The Chinese University of Hong Kong, Hong Kong, China; 4 Royal Holloway University of London, London, UK; 5 La Trobe University, Melbourne, Australia

**Keywords:** Human behaviour, Public health

## Abstract

Understanding factors driving vaccine hesitancy is crucial to vaccination success. We surveyed adults (N = 2510) from February to March 2021 across five sites (Australia = 502, Germany = 516, Hong Kong = 445, UK = 512, USA = 535) using a cross-sectional design and stratified quota sampling for age, sex, and education. We assessed willingness to take a vaccine and a comprehensive set of putative predictors. Predictive power was analysed with a machine learning algorithm. Only 57.4% of the participants indicated that they would definitely or probably get vaccinated. A parsimonious machine learning model could identify vaccine hesitancy with high accuracy (i.e. 82% sensitivity and 79–82% specificity) using 12 variables only. The most relevant predictors were vaccination conspiracy beliefs, various paranoid concerns related to the pandemic, a general conspiracy mentality, COVID anxiety, high perceived risk of infection, low perceived social rank, lower age, lower income, and higher population density. Campaigns seeking to increase vaccine uptake need to take mistrust as the main driver of vaccine hesitancy into account.

## Introduction

As COVID-19 vaccines are being rolled out, success of the vaccination crucially depends on a sufficient proportion of the population accepting a vaccine. Numerous studies have already investigated *putative* vaccine acceptance by asking people whether they would be willing to accept a COVID-19 vaccine if it were offered to them. Vaccine willingness rates vary around 65–75% of the population in most of the surveyed countries^1^. The few multi-national studies to date indicate considerable between country variance^2–4^. Even within the group of high income countries, which are now in the process of offering vaccines to all their citizens, the acceptance rates have been found to vary, with UK citizens showing particularly high vaccine willingness, Germans being more hesitant^3,4^ and particularly low rates in Hong Kong^5^. Overall, however, it is clear that fewer people are willing to take a vaccine than required for sufficient population immunity^6–8^.

To better understand the factors driving vaccine hesitancy^9^, several studies have assessed the putative predictors of COVID-19 vaccine willingness versus hesitancy. Higher vaccine willingness was found to correlate with a higher COVID-19-risk-perception^2,10,11^, whereas vaccine hesitancy correlated with vaccine safety and efficacy concerns^2,4,5,12–14^. Sociodemographic variables associated with hesitancy were younger age, female gender, lower income, lower education, unemployment, and migrant status in many of the studies^2–5,10,12,14,15^. Further predictors were extreme political views^16^, higher social media consumption^10,11,17^, mistrust of the government, research, and the medical profession^3,11,12,17^, general and COVID-19-specific conspiracy beliefs^10,12,17^, and paranoid ideation^17^.

Thus, some of the driving factors of COVID-19 vaccine hesitancy identified so far (i.e., sociodemographic factors, risk perception, trust in vaccine-safety) match those found for vaccine hesitancy in general^18,19^. Beyond those factors, the studies point to the relevance of factors indicative of a more fundamental mistrust, including mistrust of mainstream media and politics, conspiracy beliefs, and paranoid ideation.

However, we do not know how well these putative driving factors perform in predicting vaccine hesitancy, which factors are most relevant to an optimal prediction, or whether an optimal prediction in one country can be generalized to other countries. Identifying a globally stable algorithm to predict vaccine hesitancy based on a limited set of variables would provide an immensely helpful basis for targeted interventions to increase vaccine willingness. Thus, an important next step would be to probe for and optimize the prediction of vaccine willingness in a multi-national survey on the basis of variables identified as relevant so far. This could be done by using machine learning algorithms that are able to capture the complex relationships and interactions between variables^20^.

Also, given the relevance of mistrust, it seems promising to place more focus on this construct in relation to vaccine willingness. This could be done by including a more fine-grained assessment of mistrust related variables along with predictors of mistrust that have been identified in clinical research on paranoia. These include social marginalization and adversity (e.g. having a minority status or interpersonal traumatization), and negative generalized beliefs about oneself, other people, and one’s position in society^21^.

The present comprehensive multi-national survey included quota samples from five high-income sites in the early phases of vaccine rollout and addressed the following three aims:

1. to assess the prevalence of COVID-19 vaccine willingness across sites;
2. to replicate previous research on the correlates of vaccine willingness and identify the key factors driving vaccine hesitancy. To this aim, we used regression analyses to test whether COVID-19 vaccine willingness is predicted by (a) sociodemographic variables including those indicative of social marginalization, (b) perception of COVID risk, (c) political orientation and preferred types of information sources, (d) specific mistrust (i.e., vaccine conspiracy beliefs, pandemic-related paranoid ideation), (e) general mistrust (i.e., conspiracy mentality, general paranoid ideation), (f) social adversity, and (g) generalized beliefs about the self, others, and ones’ own social rank;
3. to identify vaccination hesitant people accurately based on a limited set of variables in order to provide targeted interventions to the right individuals. To this aim, we changed our focus from explanatory regression analysis to optimizing prediction. We used a machine learning model to probe for the optimum prediction accuracy for vaccine hesitancy and to find a parsimonious model based on a selection of common global predictors. We also explored the stability of the most promising predictive model across sites.

## Results

### Sample characteristics

Sample characteristics for the full sample and the individual sites are presented in Table 1.

**Table 1:** Participant flow and sociodemographic details across samples.

	UK	USA	AU	GE	HK	Total
**Participant flow**						
Participants approached for the survey	2725	1790	3209	3456	1673	12853NA
	985	536	NA	645	524	NA
Participants who completed surveys and passed attention checks	512	535	502	516	445	2510
Age, M years (SD)	41.91 (14.87)	47.65 (17.05)	44.75 (17.55)	42.00 (13.79)	39.64 (13.57)	43.32 (15.73)
**Gender (%)**						
Male Female Genderqueer Transmale/female Other	47.1% 52.7% 0% 0% 0%	46.4% 52.7% 0.2% 0.4% 0.4%	48.2% 50.8% 0.2% 0.2% 0.4%	49.2% 50.0% 0.6% 0.2% 0%	43.1% 56.6% 0% 0.2% 0%	46.9% 52.5% 0.2% 0.1% 0.2%
**Size of current home city**						
< 100.000 people	36.1%	37.6%	19.3%	55.4%	0.9%	30.8%
Up to 500.000 people	28.9%	19.6%	16.1%	20.2%	5.2%	18.4%
Up to 1 million people	8.4%	10.1%	12.5%	10.1%	3.6%	9.1%
Up to 5 million people	3.7%	8.8%	25.7%	11.2%	1.3%	10.3%
Up to 10 million people	4.9%	5.6%	10.2%	0.6%	83.8%	19.2%
Over to 10 million people	4.9%	4.7%	1.8%	0.6%	2.5%	2.9%
Unknown	13.1%	13.6%	14.3%	1.9%	2.7%	9.3%
**Educational level**						
Primary Secondary or equivalent A-level or equivalent Bachelor degree Masters degree PhD or equivalent	0.4% 19.7% 38.3% 30.3% 9.4% 2.0%	5.2% 0.0% 34.4% 46.7% 11.0% 2.6%	0.8% 15.5% 49.2% 28.9% 4.6% 1.0%	0.4% 59.7% 12.8% 11.4% 14.5% 1.2%	2.5% 28.8% 18.2% 39.8% 10.1% 0.7%	1.9% 24.5% 30.8% 31.3% 10.0% 1.5%
**Annual income**						
Under £18,500 £18,500–£36,999 £37,000–£55,999 £56,000–£74,999 £75,000–£92,999 £93,000–£111,999 £112,000 +	15.6% 39.8% 23.6% 11.5% 4.7% 2.1% 2.5%	26.7% 25% 16.1% 10.1% 6.9% 7.5% 7.7%	22.9% 27.1% 13.3% 13.3% 12.4% 7.4% 3.6%	20.9% 28.3% 23.4% 14.7% 6.2% 3.3% 3.1%	8.5% 22.2% 28.8% 11.7% 13.9% 8.3% 6.5%	19.3% 28.6% 20.8% 12.3% 8.6% 5.7% 4.7%
**Employment status**						
Full time Part time Retired Unemployed (looking) Military Unemployed (not looking) Home keeper/carer Disabled Training/school	50.4% 20.7% 10.4% 4.9% 0.0% 2.0% 5.7% 1.6% 4.3%	40.9% 8.8% 0% 4.9% 0.0% 22.1% 9.2% 4.7% 8.2%	41.8% 13.9% 16.9% 7.4% 0.0% 2.8% 7.2% 6.0% 4.0%	50.2% 17.6% 8.7% 6.2% 0.2% 1.7% 4.5% 2.5% 8.3%	74.4% 9.7% 3.6% 1.6% 0.0% 0.7% 1.3% 0.0% 8.8%	50.9% 14.2% 7.9% 5.1% 0.4% 6.1% 5.7% 3.0% 6.7%
Migrant status	12.7%	5.4%	15.9%	7.0%	5.4%	9.3%
**Minority status**						
Sexual orientation/identity	11.9%	9.9%	11.0%	10.3%	10.1%	10.6%
Ethnic minority/skin colour	11.7%	10.1%	11.4%	5.6%	8.3%	9.4%
Minority religion/belief	8.6%	12.1%	11.8%	8.9%	9.2%	10.2%
Physical disability	9.0%	15.0%	16.3%	11.8%	8.8%	12.3%
Visible physical condition	13.1%	17.8%	16.5%	22.1%	23.8%	18.5%
Part of ≥ 1 minority	37.7%	41.5%	45.0%	39.7%	36.6%	40.2%
Mental health diagnosis	12.3%	22.4%	41.8%	20.0%	7.2%	21.0%

An overview of the descriptive values for the remaining predictor variables can be found in supplement 5.

### Prevalence of vaccine willingness

Table 2 shows vaccine willingness across sites. Only 57.4% of all participants indicated that they would definitely or probably get vaccinated. The distribution of the answers varied considerably between sites. In the USA and Germany, a bi-modal distribution of answers with peaks in definite willingness and definite rejection of the vaccine were found. In the UK and Australia, by contrast, there were skewed distributions with most participants indicating definite willingness. Finally, most participants in the Hong Kong sample answered in the mid-category indicating possible willingness for vaccination, with few participants responding with definite acceptance or rejection. An ANOVA of vaccine willingness showed a significant effect of site (F(4,2505) = 59.65, p < 0.001, η^2^ = 0.087). Bonferroni-corrected post hoc comparisons indicated a higher mean willingness in the UK than in all other sites (USA: *T* = 8.61, *p*_*corr*_ < 0.001, d = 0.533, Australia: *T* = 6.94, *p*_*corr*_ < 0.001, *d* = 0.436, Germany: *T* = 9.14, *p*_*corr*_ < 0.001, *d* = 0.570, Hong Kong: *T* = 18.11, *p*_*corr*_ < 0.001, *d* = 1.173) and in Hong Kong a lower mean willingness than all other sites (USA: *T* = 6.68, *p*_*corr*_ < 0.001, *d* = 0.429, Australia: *T* = 9.53, *p*_*corr*_ < 0.001, *d* = 0.621, Germany: *T* = 6.89, *p*_*corr*_ < 0.001, *d* = 0.445).

**Table 2:** Distribution of vaccine willingness across countries.

Answer category	Definitely rejecting vaccination if offered		Probably rejecting vaccination if offered		Possibly taking vaccination if offered		Probably taking vaccination if offered		Definitely taking vaccination if offered	
Dichotomized category	Vaccination Hesitancy				NA		Vaccination willingness			
	*n*	%	*n*	%	*n*	%	*n*	%	*n*	%
UK	28	5.5	40	7.8	40	7.8	59	11.5	345	67.4
USA	114	21.3	52	9.7	61	11.4	64	12.0	244	45.6
Australia	58	11.6	67	13.3	55	11.0	110	21.9	212	42.2
Germany	83	16.1	63	12.2	88	17.1	79	15.3	203	39.3
Hong Kong	44	9.9	125	28.1	150	33.8	83	18.7	43	9.7
Total	327	13.0	347	13.8	394	15.7	395	15.7	1047	41.7

### Prediction of vaccine willingness using regression

As can be seen in Table 3 (left column, correlation), most variables showed significant correlations with vaccine willingness. The strongest associations were found for COVID anxiety (positive association), vaccine conspiracy beliefs, pandemic conspiracy beliefs, and general conspiracy mentality (all negative associations). A follow-up-calculation of correlations by site (see supplement 2) showed that the negative association between vaccine willingness and vaccine conspiracy beliefs (− 0.68 ≤ *r* ≤  − 0.41), pandemic conspiracy beliefs (− 0.53 ≤ *r* ≤  − 0.21), general conspiracy mentality (− 0.36 ≤ *r* ≤  − 0.27), and gender (− 0.16 ≤ *r* ≤  − 0.10), as well as the positive association with positive beliefs about others (0.13 ≤ *r* ≤ 0.24) could be found within each site. Furthermore, the association with age, education, income, risk-perception variables, primary news source and the remaining generalized beliefs -variables were found in the majority of the sites. Notably, none of the perception of COVID risk variables showed a significant correlation with vaccine willingness in the Hong Kong sample. Finally, we found the following site-specific correlation in the opposite direction when compared to the full sample: size of current home city (USA: *r* = 0.17, *p* < 0.001), right-wing political orientation (Hong Kong: *r* = 0.25, *p* < 0.001), and higher pandemic paranoia global score (Australia: *r* = 0.11, *p* = 0.016).

**Table 3:** Correlation and multifactorial logistic regression analyses predicting vaccine willingness vs. hesitancy (n = 2116).

	Correlation		Regressions per variable cluster			Regression with all variables		
	*r*	*p*	*OR*	*Z*	*p*	*OR*	*Z*	*p*
**Socio-demographic data**								
Age	0.170***	< 0.001	1.537***	8.27	< 0.001	1.555***	5.26	< 0.001
Gender (0 = male, 1 = female)^a^	−0.133***	< 0.001	0.637***	−4.50	< 0.001	0.602***	−3.42	0.001
Size of current home city	−0.073***	< 0.001	0.796***	−4.38	< 0.001	0.688***	−4.88	< 0.001
Educational level (0 = “ ≥ A-level”, 1 = primary/secondary)^b^	−0.101***	< 0.001	0.713**	−3.01	0.003	0.632**	−2.86	0.004
Annual income	0.132***	< 0.001	1.417***	6.05	< 0.001	1.171	1.91	0.056
Employment status (0 = “working”, 1 = “not working”)	−0.039	0.074	0.725*	−2.27	0.023	0.801	−1.07	0.285
Migrant status (0 = “no” vs. 1 = “yes”)	0.023	0.285	1.168	0.90	0.368	1.679*	2.13	0.034
Minority status (0 = “no” vs. 1 = “yes”)	0.007	0.764	1.586***	2.81	0.005	1.272	1.07	0.285
Number of minority group memberships	−0.024	0.27	0.868	−1.81	0.070	1.002	0.02	0.987
Mental health diagnosis	0.014	0.514	0.941	−0.50	0.620	0.662*	−2.18	0.029
**Perception of COVID risk**								
COVID anxiety	0.237***	< 0.001	1.454***	6.46	< 0.001	1.266**	2.67	0.007
COVID in family members/friends	0.105***	< 0.001	1.405**	2.93	0.003	1.418*	2.08	0.037
Perceived risk of infection	0.194***	< 0.001	1.210**	2.97	0.003	1.393***	3.60	< 0.001
Expected consequences of infection	0.151***	< 0.001	1.039	0.65	0.516	0.911	−1.06	0.292
**Political mindedness**								
Political orientation (higher values = more right wing orientation)	−0.101***	< 0.001	0.792***	−5.03	< 0.001	0.850*	−2.28	0.022
Primary source of information (higher values = more social media)	−0.142***	< 0.001	0.723***	−6.77	< 0.001	1.119	1.49	0.137
**Specific mistrust**								
Pandemic persecutory threat (PPS)	0.059**	0.006	2.464***	10.90	< 0.001	1.844***	5.35	< 0.001
Pandemic paranoid conspiracy (PPS)	−0.389***	< 0.001	0.601***	−4.88	< 0.001	0.615***	−4.15	< 0.001
Pandemic interpersonal mistrust (PPS)	−0.110***	< 0.001	1.801***	7.21	< 0.001	1.746***	5.73	< 0.001
Pandemic paranoia global score (PPS)	−0.052*	0.017	-	-	-	-		
Vaccine conspiracy beliefs	−0.559***	< 0.001	0.167***	−18.23	< 0.001	0.159***	−15.65	< 0.001
**General mistrust**								
Ideas of reference (RGPTS)	−0.046*	0.033	1.051	0.53	0.596	1.371*	2.21	0.027
Paranoid ideation (RGPTS)	−0.042	0.056	1.106	1.08	0.281	1.013	0.09	0.932
General conspiracy mentality (CMQ)	−0.351***	< 0.001	0.402***	−15.23	< 0.001	1.035	0.36	0.716
**Social adversity**								
Traumatic emotional neglect	0.082***	< 0.001	1.088	0.72	0.469	0.905	−0.57	0.571
Traumatic psychological abuse	0.122***	< 0.001	1.632***	3.80	< 0.001	1.245	1.16	0.245
Traumatic physical abuse	0.047*	0.036	0.846	−1.34	0.181	0.803	−1.15	0.250
Traumatic sexual abuse	0.083***	< 0.001	1.249	1.89	0.058	1.405	1.89	0.059
**Generalized beliefs (self, others, own social rank)**								
Negative beliefs about self (BCSS)	−0.097***	< 0.001	1.014	0.22	0.834	0.955	−0.47	0.640
Negative beliefs about others (BCSS)	−0.152***	< 0.001	0.798***	−4.20	< 0.001	0.853	−1.89	0.059
Positive beliefs about self (BCSS)	0.073***	< 0.001	0.831**	−2.58	0.010	0.863	−1.48	0.139
Positive beliefs about others (BCSS)	0.182***	< 0.001	1.489***	6.64	< 0.001	1.438***	4.30	< 0.001
Perceived social rank (SCS)	0.107***	< 0.001	1.132	1.83	0.068	1.143	1.34	0.182

(a) To avoid bias due to low cell counts the variables sex and gender were combined into a dichotomized variable to reflect the gender a participants most likely reads as at present (e.g. a person describing their sex as male and their gender as trans/female was labeled as female; a person describing their sex as female and their gender as other was labeled as female) leading to a recoding for 16 participants (0.63%); (b) education level was dichotomized with GCSE or lower categorized as low educational level and everything else as high educational level.

*PPS *pandemic paranoia scale, *CMQ *conspiracy mentality questionnaire, *RGTPS* revised green paranoid thoughts scale, *BCSS *brief core schema scales, *SCS* social comparison scale.

Among cluster-specific logistic regression models (see Table 4), the *specific mistrust* model yielded the highest total accuracy (*TAC* = 0.84, *Nagelkerke's R²* = 0.54), followed by the *general mistrust* model (*TAC* = 0.73, *Nagelkerke's R²* = 0.18) and the extended *socio-demographic* (TAC = 0.70, *Nagelkerke's R²* = 0.12) and *perception of COVID risk model* (*TAC* = 0.70, *Nagelkerke's R²* = 0.09). The social adversity model did not provide any additional accuracy beyond classifying all participants into the vaccine willingness group (*TAC* = 0.68). The combined regression model with all variables showed a total accuracy of 0.85 (*Nagelkerke's R²* = 0.65). For all logistic regression models, accuracy was driven by high sensitivity (i.e., correctly identifying vaccine willingness), but comparatively low specificity (i.e., correctly identifying vaccine hesitancy; see Table 4).

**Table 4:** Accuracy of the logistic regression and machine learning model (ML cross-validation using leave-one-site-out and the leave-one-person out method).

Included/added variables	Sensitivity (willingness)	PPV	Specificity (hesitancy)	NPV	BAC	TAC
**Logistic regression models**						
Socio-demographic data	0.93	0.72	0.21	0.58	0.57	0.70
Perception of COVID risk	0.94	0.72	0.20	0.60	0.57	0.70
Political mindedness	0.98	0.69	0.04	0.56	0.51	0.68
Specific mistrust	0.92	0.85	0.66	0.80	0.79	0.84
General mistrust	0.92	0.74	0.32	0.66	0.62	0.73
Social adversity	1.00	0.68	0.00	-	0.50	0.68
Generalized beliefs	0.95	0.70	0.11	0.54	0.53	0.69
All variables included	0.92	0.87	0.70	0.81	0.81	0.85
**Machine learning models (leave-one-site-out cross validation)**						
All variables included	0.82	0.89	0.78	0.67	0.80	0.81
Vaccination conspiracy beliefs excluded	0.78	0.84	0.68	0.59	0.73	0.74
Specific/general mistrust excluded	0.70	0.78	0.59	0.47	0.65	0.66
12 best variables	0.82	0.89	0.79	0.67	0.81	0.81
7 best variables	0.80	0.89	0.78	0.65	0.79	0.80
**Machine learning models (leave-one-person-out cross validation)**						
All variables included	0.82	0.91	0.82	0.68	0.82	0.82
Vaccination conspiracy beliefs excluded	0.82	0.86	0.71	0.65	0.77	0.79
Specific/general mistrust excluded	0.68	0.82	0.69	0.50	0.69	0.68
12 best variables	0.82	0.91	0.82	0.68	0.82	0.82
7 best variables	0.81	0.91	0.84	0.68	0.83	0.82

*PPV* positive predictive value (the frequency true vaccination willing among people all predicted to be vaccination willing), *NPV* negative predictive value (the frequency true vaccination hesitant among all people predicted to be vaccination hesitant, *BAC* balanced accuracy (the average of sensitivity and specificity), *TAC* total unweighted accuracy.

### Prediction of vaccine willingness using machine learning

**Figure 1:**
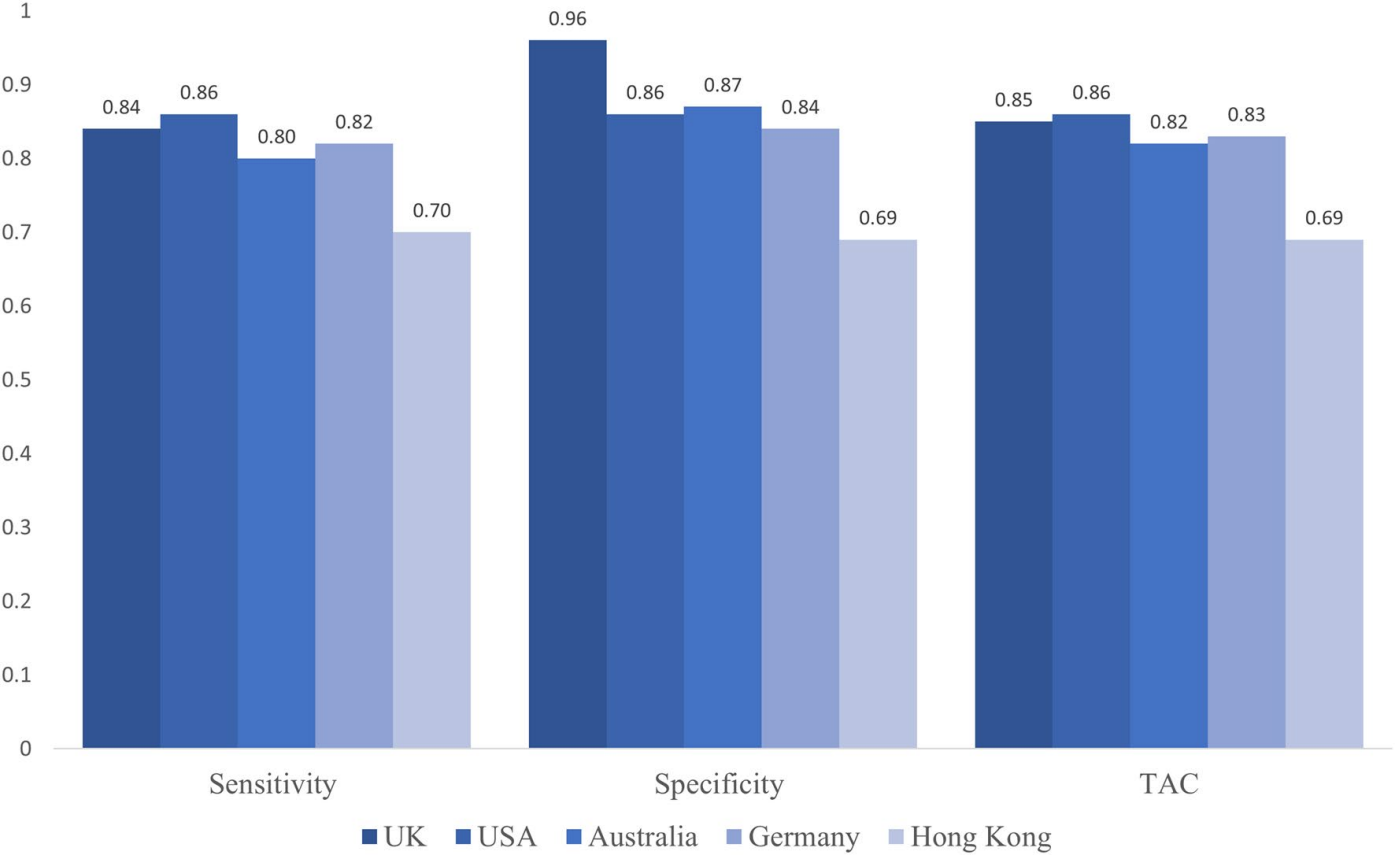
Accuracy of the leave-one-person-out cross validation of the all-variables-machine-learning model by site.

For both cross-validation methods, a machine learning model showed high balanced accuracy (see Table 4; details on the hyperparameter tuning results are provided in supplement 3). As can be seen, the full model was able to correctly classify 82% of the participants who were willing to get vaccinated (i.e., sensitivity) that were left out for cross validation in the leave-one-site-out and leave-one-person-out validation, respectively. Furthermore, the model was able to correctly identify most participants who indicated an unwillingness to get vaccinated (i.e., specificity). However, in the leave-one-site-out cross validation, the full model showed somewhat lower specificity (78%) than in the leave-one-person-out model (82%). Furthermore, splitting the accuracy scores of the leave-one-person-out cross validation by site showed equally high sensitivity, specificity and total accuracy (all > 80%) for the UK, USA, Australia, and Germany, whereas Hong Kong showed decreased sensitivity (70%) and specificity (69%; see Fig. 1).

**Figure 2:**
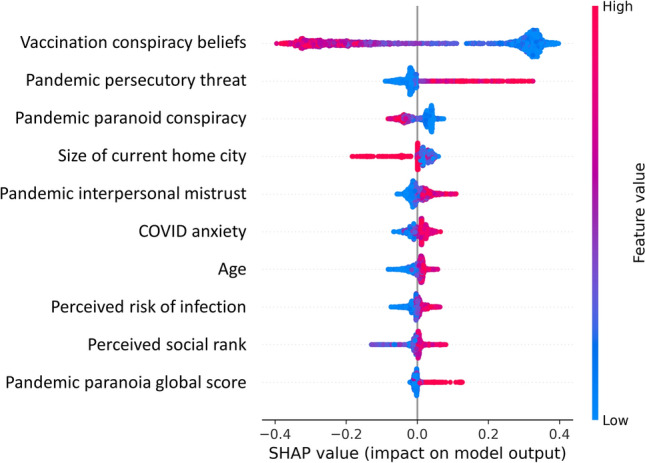
Beeswarm plot of SHAP-calculation for the ten highest ranking variables. Variables are sorted by their mean absolute SHAP value in descending order with most important variables at the top. Each dot corresponds to one person in the study. The beeswarm plot shows how the different variable expressions of each person affect the prediction of the ML model towards vaccine willingness. Positive SHAP values indicate a change in the expected model prediction towards vaccine willingness. The plot is based on the ML model with all variables included and leave-one-site-out-cross validation.

Feature importance analyses (see Table 5) and SHAP (see Fig. 2) converged on the majority of most important variables. Vaccination conspiracy beliefs was the most informative variable in both models, with a decrease in accuracy of 23.8% when this variable was permuted. The remaining nine of the top ten informative variables in feature importance included pandemic specific mistrust variables, general conspiracy mentality, social rank, COVID anxiety, perceived risk of infection, age, and income (see Table 5, left column). SHAP showed overlap with feature importance analysis in 8 variables, the only differences were the inclusion of pandemic conspiracy beliefs (instead of general conspiracy beliefs), and size of current home city (but not income).

**Table 5:** Variable importance for the ten highest ranking variables across each model based permutation feature importance.

Rank	Model					
	All variables included		Vaccination conspiracy beliefs excluded from model		Specific/general mistrust excluded from model	
	Variable name	Δacc	Variable name	Δacc	Variable name	Δacc
1	Vaccination conspiracy beliefs	0.238	Pandemic paranoid conspiracy	0.139	COVID anxiety	0.032
2	Pandemic persecutory threat	0.037	Pandemic persecutory threat	0.033	Age	0.017
3	Pandemic paranoia global score	0.012	Pandemic interpersonal mistrust	0.012	Positive beliefs about others	0.008
4	Low social rank	0.012	Low social rank	0.007	Primary source of information	0.007
5	Pandemic interpersonal mistrust	0.006	Pandemic paranoia global score	0.006	Gender	0.007
6	COVID anxiety	0.006	Age	0.006	Negative beliefs about others	0.006
7	Age	0.006	COVID anxiety	0.006	Perceived risk of infection	0.005
8	Perceived risk of infection	0.005	Positive beliefs about others	0.005	Traumatic psychological abuse	0.001
9	Annual income	0.002	General conspiracy mentality	0.005	Migrant status	0.000
10	General conspiracy mentality	0.001	Ideas of reference (RGPTS)	0.004	Traumatic emotional neglect	0.000

*Δacc* values indicate the mean decrease in accuracy over ten permutations of the respective variable.

Calculating a new model without vaccine conspiracy beliefs only resulted in a slight drop in accuracy with the specificity being more affected than the sensitivity. In this model, COVID specific and general conspiracy beliefs increased in feature importance, and positive beliefs in oneself and others moved up into the list of the top ten most relevant variables (see Table 5, mid column). Leaving out all specific and general mistrust variables led to a considerable drop in accuracy with the most informative variable in the model now being COVID anxiety followed by a mix of variables from all remaining clusters (see Table 5, right column).

The calculation of two parsimonious models was based on the combination of the ten and five most important variables from feature importance and SHAP (Table 5, left column, and Fig. 2). Differences in the ranking between both methods lead to the inclusion of twelve and seven variables, respectively. Cross-validation yielded the same accuracy as the full model (twelve variables) or a minimal decrease in accuracy (seven variables; Table 4).

### Exploratory analyses including vaccination-indecisive participants

In order to explore whether our model can be extended to people who indicated that they would possibly take the vaccine (mid-category), we re-ran the random-forest classification based on all predictors twice, first with the mid-category added to the group of vaccine-hesitant participants (model 1) and then with the mid-category added as a separate category of indecisive participants (model 2). Both models performed poorer than the corresponding main analyses model (model 1: BAC = 0.73–0.76, model 2: BAC = 0.55–0.59). Specifically, the results from the multiclass-RF in model showed that whereas correct classification remained fairly high for willingness (Recall: 0.67–0.69) and hesitancy (Recall: 0.65), the model largely failed to correctly allocate indecisive participants (Recall: 0.34–0.42, see supplement 6).

## Discussion

Surprisingly, only 57.4% of the total sample indicated that they would definitely or probably get vaccinated, which is a somewhat lower percentage than the 65–75% identified previously^3,4^. The lower rates might stem from the fact that vaccine side effects were receiving a lot of media attention during the assessment period^22^, and online misinformation on vaccination was rocketing^23^. Thus, it seems that vaccine willingness is not necessarily stable over time. However, differences between countries also need to be considered: Corresponding with the two previous multinational studies, we found the UK to show comparably high vaccine willingness and lower rates for the US and Germany^3,4^. Interestingly, the distributions of the willingness scale also differed between sites. The USA and Germany tended more to the extremes (i.e., clear refusal or willingness), whereas participants from Hong Kong showed more indecisiveness, which may be partly explicable by safety and effectiveness concerns associated with specific vaccines being offered in Hong Kong^5^.

In terms of predicting vaccine willingness in logistic regression, we could confirm most of the included factors that were delineated from previous research or from clinical models of paranoia. The clearest finding was the strong predictive value of specific mistrust, which correctly identified 84% participants as vaccine hesitant or willing to get vaccinated. This translates to an explained variance of 54%, exceeding previous associations between vaccine hesitancy and mistrust^12^. Within the variables indicative of mistrust, the strongest associations with vaccine willingness were found for vaccine conspiracy beliefs and pandemic paranoid conspiracy beliefs. Interestingly however, one type of mistrust within this group, namely *not trusting others* to comply with the COVID measures, correlated with higher willingness to get vaccinated, suggesting that pandemic mistrust is a multi-faceted construct, with facets that are associated with opposing behavioral responses. The next best predictors were variables indicative of more general mistrust, particularly general conspiracy mentality but also general paranoid ideation (total accuracy 73%, pseudo-R² 18%). In terms of demographics, we could confirm the associations with vaccine hesitancy from prior studies (e.g. younger age, female gender, unemployment, living in a larger city), except for migrant or minority status. Finally, it needs noting that the putative predictors varied in the stability of their association with vaccine hesitancy across sites. Whereas all types of conspiracy beliefs, positive beliefs about others, gender and (to a lesser degree) age showed consistent associations with vaccine hesitancy in all sites, results for other variables were more heterogeneous. For example, living in a larger city was associated with vaccine hesitancy in the UK, but with vaccine willingness in the USA. A right-wing political orientation correlated with hesitancy in the UK, USA, Australia, and Germany, but with willingness in Hong Kong. This pattern of findings suggests that while some putative driving factors of vaccine hesitancy, such as conspiracy beliefs, could be common global factors, others seem to depend on the regional context.

Using machine learning, we were able to achieve a high prediction accuracy with balanced levels of sensitivity and specificity and to find a parsimonious model with a sensitivity of 82% and a specificity of 78–82%, depending on the type of cross-validation. This model confirmed the high predictive value of vaccine conspiracy beliefs and other indicators of specific mistrust, but also used the perception of social rank, COVID anxiety and perceived risk of infection, as well as demographic variables to optimize its prediction. Despite the high relevance of the vaccine conspiracy beliefs, they were not essential to good prediction and could be compensated for by putting more weight on COVID specific and general conspiracy beliefs, resulting in almost as good prediction accuracy. In contrast, models that were not fed with any mistrust variables performed poorly.

There was no drastic drop in the models’ prediction accuracy depending on the method of cross-validation. Accurate test predictions were found both when we trained the model on all participants but one and tested it on the remaining participant and when we trained it on the four sites before testing it on the fifth site. Accuracy scores for the individual sites revealed considerable variation in the machine learning model’s performance, with a comparatively low accuracy for the Hong Kong site in particular. It needs noting that in the Hong Kong sample some correlations diverged from the total sample. Namely, there were no associations between perception of COVID risk variables and vaccine willingness. The general prediction model, however, relied to a certain degree on perception of COVID risk variables such as COVID anxiety. Thus, the drop in accuracy could be explained by the difference in associations between risk perception and vaccine willingness. Possibly, the decision to vaccinate oneself in a more collectivistic culture such as Hong Kong^24^ is driven by factors of reducing the risk within one’s immediate environment rather than just the risk for oneself^25^. Another explanation is that the number of indecisive participants was particularly large in the Hong Kong sample and the generalizability of our model is limited to settings characterized by polarized opinion about vaccination (i.e., the predominant public dispute on vaccination in Western societies).

A limitation of the study is that although respondents included diverse samples of the sites’ adult general populations in terms of age, sex, and educational level, they are unlikely to be fully representative, limiting the generalizability to the population. The percentages of those who declined participation varied across sites and the reasons for declining as well as the demographic characteristics of dropouts are unknown. This needs to be kept in mind especially when interpreting the point estimate of vaccine willingness or mean values and the distribution of predictors. Also, the sites do not reflect the global variability in cultures, thus the status of variables such as conspiracy beliefs as common global predictors requires further validation in more heterogeneous samples of countries. Another limitation is the cross-sectional nature of the design. Although the causal interpretation that mistrust is driving vaccine refusal is tempting, we need to bear in mind that the opposite direction (e.g. vaccine conspiracy could be a post hoc rationalisation of not wanting a vaccine for other reasons) is also a possibility given that we only know that both co-vary at this point. Finally, it needs noting that vaccine willingness may not accurately predict actual vaccine uptake, albeit the low willingness we found seems to be confirmed by the hesitant uptake of the vaccines currently being rolled out^26^. The extent to which the machine learning model predicting vaccine willingness holds up for predicting actual vaccine intake is also an issue for future research.

In sum, we found that by using only twelve variables (the combined most important variables from permutation feature importance and SHAP) we were able to achieve an 82% accuracy in predicting vaccine hesitancy, with the most crucial factors being vaccination conspiracy beliefs and a lack of confidence in governments, companies, and organizations in handling the pandemic (i.e., pandemic conspiracy beliefs). The reasons for this type of societal mistrust are manifold^27^, but have been found to include both individual societal experiences, such as downward social mobility^28^ and the perception of past and present institutional misperformance^29^. Institutions that do not perform well, be it by incompetence or elite misbehaviour and corruption, tend to generate distrust^30^. People are more likely to attend to and believe information that aligns with their expectations (confirmation bias)^31^. Conspiracy theories align well with negative expectations that have resulted from previous experiences, rendering them more likely to be believed. The high predictive value of vaccine conspiracy beliefs clearly corroborates the efforts towards strategic approaches to detect and mitigate the impact of anti-vaccine activities on social media^23,32,33^. However, given that our machine learning algorithm performed almost as well by relying solely on other indicators of COVID specific, merely reducing or contradicting vaccine conspiracy information might not be sufficient. Publicly provided vaccine information needs to take these other types of mistrust into account. This could be done by providing information on the safety and effectiveness of the vaccine in a way that enables the recipients to judge its validity for themselves and by complementing information campaigns by policies aimed at regaining peoples’ trust in politicians, industry, science, and the medical profession.

## Methods

### Design & procedure

The design was a cross-sectional online-survey conducted in Hong Kong, Australia, USA, United Kingdom, and Germany. The survey was programmed using the online-survey platform Qualtrics. Participants were recruited using stratified quota sampling to ensure that each sample was quota sampled at each site based on sex, age, and educational attainment. No further eligibility criteria were applied. Data were collected between February and March 2021. We aimed for a sample size of 2500 taking into account the stratification and number of sites, the large number of predictors, and expected small effect sizes of some of the putative predictors. The survey took 25 min in total, beginning with informed consent, followed by socio-demographic assessment and the questionnaire battery, of which further details have been reported elsewhere^34^. To prevent missing data, participants were required to respond to all questions on each page before being able to continue. The missing data was thus minimal and resulted from initial software errors (Missings were present for: “perceived risk of infection”: 0.2%, n = 7, “preferred sources of information”: 2.8%, n = 72, and “social adversity”: 0.1%, n = 3) or from a “don’t know” answering option (“size of the current home city”: 9.3%, n = 234). Missing values in these independent variables were imputed prior to the analyses using the k-Nearest-Neighbor algorithm, with each missing value being imputed based on the unweighted mean of 3 related cases. Participants who failed any of the attention checks, took shorter than half of the median completion time, or showed patterns of machine responses or duplicate patterns of response were excluded.

All procedures were approved by each of the ethics committees of the institutions involved (i.e., (1) Royal Holloway, University of London Research Ethics Committee, Reference No. 2368, (2) Care New England—Butler Hospital Institutional Review Board, Reference No. 202012–002, (3) La Trobe University Human Research Ethics Committee, Application No. HEC21012, (4) Local Ethics Committee, Universität Hamburg, Application No. 2020_346, and (5) The Chinese University of Hong Kong Survey and Behavioural Research Ethics Committee Reference No. SBRE-20–233). This manuscript follows the STROBE statement for reporting of observational studies.

### Role of funding source

There was no funding source for this study.

### Measures

*Willingness to be vaccinated* for COVID-19 was assessed with the following item: “If a COVID-19 vaccine was offered to you now, would you accept it?” The item was rated on a scale from 1 = “Definitely not” to 5 = “Yes, definitely’” adapted from Wong and colleagues^35^.

### Sociodemographic data and related questions

Sociodemographic variables included age, sex assigned at birth, and current gender (options: “male”, “female”, “trans-male”, “trans-female”, “genderqueer”, and “other”), size of the current home city (rated in six categories form ≤ 100.000 to ≥ 10.000.000), highest educational degree achieved (rated in nine categories from elementary school degree to PhD), annual income (seven categories from “under £18,500/US$24,999/18,000€” to “above £112,000/US$150,000/109,000€”), employment status over the last year (nine categories), migrant status, minority status (five categories, each rated as present or absent), and having a mental-health diagnosis.

*Perception of COVID risk* variables included (1) COVID-19 anxiety, (2) personal experiences with COVID-19 in family members or friends, (3) perceived risk of infection, and (4) expected consequences of an infection. Following Shevlin et al.^36^ COVID-19 anxiety was assessed using the question “How anxious are you about the coronavirus COVID-19 pandemic?” for which participants were provided with a ‘slider’ to indicate their degree of anxiety with 0 = “not at all worried” and 100 = “very worried”. Personal experiences with COVID-19 in family members or friends were assessed by the following item: “Someone who is close to me has had a COVID-19 virus infection confirmed by a doctor” rated with 1 = “yes” 0 = “no”. Perceived risk of a COVID-19 infection was assessed with the item: “What do you think is your personal percentage risk of being infected with the COVID-19 virus over the following time periods?” rated from 1 = “no risk” to 11 = “great risk” for each time period (“the next month”, “the next three months”, and “the next six months”). Similarly, the expected consequence of an infection was assessed with “How bad do you think would be the consequences of you being infected with the COVID-19 virus over the following time periods?” rated from 1 = “not too bad” to 11 = “very bad”. Mean scores of perceived risk and expected consequences were calculated.

*Political orientation* was rated from 1 = ”very left-wing” to 7 = ”very right wing” and *preferred sources of information* (“How do you find out about what is going on in the world?”) were rated from 1 = “always from mainstream media” to 5 = “always from social media”^10^.

*Specific mistrust* variables included (1) COVID-specific paranoid ideation and (2) vaccine conspiracy beliefs. COVID-specific paranoid ideation was assessed with the Pandemic Paranoia Scale^34^, a 25-item scale assessing paranoid thinking specifically related to the COVID-19 pandemic. It comprises a *pandemic paranoia global score* and the three facets *pandemic persecutory threat* (15 items, e.g.: “People are deliberately trying to pass COVID-19 to me”), *pandemic paranoid conspiracy* (six items, e.g.: “COVID-19 is a conspiracy by powerful people”), and *pandemic interpersonal mistrust* regarding health measures (four items, e.g.: “I can’t trust others to stick to the social distancing rules”). Participants answer on a scale from 0 = “not at all” to 4 = “totally”. Based on the data used for this article, Kingston et al. ^34^ reported good reliability (internal consistency: *α* = 0.90, test–retest reliability: 0.60 ≤ *r* ≤ 0.78), factorial validity, and criterion validity. For this study, the three subscales and the global score were calculated. Vaccine conspiracy beliefs were assessed by adapting the general 7-item Vaccine Conspiracy Beliefs Scale^37^, a valid one-dimensional scale with high internal consistency. The adaptation involved referring to COVID-19 vaccines specifically and using present tense (full item-list in supplement 1). Reliability in this study was *α* = 0.97.

*General mistrust* variables included paranoid ideation and general conspiracy mentality. Paranoid ideation was measured with the Revised Green Paranoid Thoughts Scale^38^. This 18-item questionnaire assesses *ideas of reference* and *persecutory ideation* over the past fortnight on two scales. Each item (e.g. “Certain individuals have had it in for me”) is rated on a scale from 0 = “not at all” to 4 = “totally”. Higher scores indicate higher levels of paranoia. Reliability in this study was *α* = 0.94 for ideas of reference and *α* = 0.96 for persecutory ideation. General conspiracy mentality was assessed with the Conspiracy Mentality Questionnaire^39^ an instrument designed to efficiently assess differences in the generic tendency to engage in conspiracist ideation within and across cultures. A one-dimensional and time-stable construct has been confirmed across several language versions. It consists of five statements (e.g. “Many very important things happen in the world, which the public is never informed about”) that are rated in terms of their likeliness on scale from 0 = “0% chance” to 11 = “100% chance”. Reliability in this study was *α* = 0.91.

*Social adversity* was screened alongside socio-demographic variables with a four item self-report questionnaire used by Jaya and colleagues^21^. The items consisted of yes/no questions covering emotional neglect, psychological abuse, physical abuse, and sexual abuse (e.g., “were you ever approached sexually against your will?”).

*Generalized beliefs about self, others, and one’s own social rank* were assessed with the Brief Core Schema Scales (BCSS)^40^ and the Social Comparison Scale (SCS)^41^. The BCSS assesses negative and positive beliefs about oneself and others on four subscales of six items, respectively (e.g., “Other people are bad”) that are rated as yes versus no. For each yes-response the degree of conviction is assessed on a scale from 1 = “no, do not believe it” to 5 = “yes, believe it totally”. Reliability for the subscales in the current study ranged from *α* = 0.85 to *α* = 0.90. The SCS consists of 11 bipolar items that ranged from 0 to 10 (e.g., inferior-superior, left out-accepted) that are rated over the past four weeks. Lower scores indicate a more negative view of the self in comparison with others. Reliability in this study was *α* = 0.95.

An extended overview of all predictors including reliability scores by site can be found in supplement 1.

### Analyses

Statistical analyses were conducted with SPSS 22^42^. For all main analyses, a dichotomized variable *vaccine willingness* (i.e. “definitely” or “probably” getting vaccinated) versus *vaccine hesitancy* (“definitely not” or “probably not” getting vaccinated) was used as dependent variable. The mid-category of “possible” willingness was left out for the main analyses for two reasons. One was that its sample size was relatively small, further complicating any efforts to balance classes in machine learning algorithms. The other was that the category could not be unambiguously sorted into the willingness or hesitancy category. This lead to a final analysed sample of n = 2116.

First, we calculated point-biserial correlations for all predictor variables. Next, we calculated multifactorial logistic regression models for each of the variable clusters (1) extended socio-demographic data, (2) perception of COVID risk, (3) political mindedness, (4) specific mistrust, (5) general mistrust (5) interpersonal trauma, and (6) beliefs about the self, others, and social rank in order to identify the most influential variables compare the accuracy of identifying vaccine willingness vs. hesitancy for each of these different predictor types. In a final regression model, all variables were entered to evaluate the overall accuracy of a regression based approach and to identify the driving factors of vaccine willingness vs. hesitancy. Metric variables were z-standardized to allow for a comparison of odds ratios. All significance tests for correlations and predictors in regression models were two-tailed tests.

Next, to further testing for optimization of prediction accuracy, we established a machine learning algorithm using all assessed variables. Calculation of machine learning models were carried out in Python 3.8.6 with the packages scikit-learn 0.23.2^43^, as well as Numpy, Pandas and imblearn. For all tested models we used random forest classifiers because the random forest algorithm can model non-linear relationships and complex interactions between variables without pre-specification. Random-forest was thus chosen as the best possible trade-off between potential complexity of the generated model (other approaches such as logistic regression or lasso/ridge regression require to pre-specify the relationship between independent and dependent variables) and practicability given our sample size (other models capable of modelling complex interactions, e.g., neural networks, require larger datasets to be accurately computed).

All ML-model calculations started with a hyperparameter tuning on a class-balanced version of the dataset first (see supplement 3 for details). Next, we chose the hyperparameter configuration that had the best testing accuracy and evaluated model performance by leave-one-site-out cross validation and by leave-one-person-out cross validation^20^. Finally, we used the calculated machine learning model to evaluate the predictive value of the individual variables. We used SHapley Additive exPlanations (SHAP^44^) and permutation feature importance^45^ (see supplement 4 for details) to estimate the importance of each variable in a given model. This allowed for the selection of the highest ranking variables to test whether subsequent smaller machine learning models that use only a small selection of questionnaires retain accuracy. Furthermore, it allowed for the elimination of the highest ranking variables/variable cluster to further explore their absolute relevance (i.e., whether they could be compensated for by other predictors).

## Supplementary Information

Supplementary Information.

## Data Availability

The study protocol, statistical analysis plan, and the machine learning code for implementation of the models will be made available on OSF immediately following publication. Agreement of the national ethic boards who approved the study will be required for any sharing of individual participant data. Aggregated data will be provided for meta-analyses upon request to the first author.
