# Supplementary material for: Taking a machine learning approach to optimize prediction of vaccine hesitancy in high income countries
Source: Sci Rep. 2022 Feb 8;12:2055. doi: 10.1038/s41598-022-05915-3 (PMC8827083; doi:10.1038/s41598-022-05915-3)
Supplement: Supplementary file 1 — Supplementary Information. [file 41598_2022_5915_MOESM1_ESM.pdf]

Taking a machine learning approach to optimize prediction of vaccine hesitancy in high income countries.

Tania M. Lincoln*, Björn Schlier, Felix Strakeljahn, Brandon A. Gaudiano, Suzanne H. So, Jessica Kingston, Eric Morris, Lyn Ellett.

Supplement 1. Extended information on questionnaires.

Items of the COVID-19 adapted version of the 7-item Vaccine Conspiracy Beliefs Scale:

- Vaccine safety data for COVID-19 is faked;
- Immunizing children and other vulnerable groups is harmful and this fact is covered up;
- Pharmaceutical companies are covering up the dangers of COVID-19 vaccines;
- People are lied to about COVID-19 vaccine effectiveness;
- Vaccine effectiveness data is probably faked;
- People are lied to about vaccine safety;
- The government are trying to cover up links between the vaccines being tested and serious health problems

Table S1

Extended overview of predictors with scoring, as well as example items, Cronbach's alpha by site

			Reliability by site (Cronbach's α)				
Variable	Scoring	Assessment/sample item	UK	USA	AUS	GER	HK
Socio-demographic data							
Age	Age in years	1 item: "What is your Age?"-	-	-	-	-	-
Gender	0="male", 1="female"	2 items (sex at birth, current gender), dichotomized	-	-	-	-	-
Size of current home city	(1="<100,000 people" , 6=">10 million people")	1 item: "How large is the population size of your city?"	-	-	-	-	-
Educational level	0="≥A-level", 1=primary/secondary	1 item "What is your highest educational degree?" (6 options, dichotomized)	-	-	-	-	-
Annual income	1="Under £18,500", 7="more than £112,000"	1 item: "What is the average yearly income of your household?"	-	-	-	-	-
Employment status	0="currently working", 1= "currently not working"	1 item: "What was your usual employment pattern during the past year?" (9 options, dichotomized)	-	-	-	-	-

Migrant status	0="no", 1="yes"	1 item: "Do you currently live in the same country as you were born in?"	-	-	-	-	-
Minority status	0="no", 1="yes"	At least one of 5 minority status questions answered with yes	-	-	-	-	-
Number of minority group memberships	Sum-score, range 0-5	5 yes/no-questions on minority group membership (e.g., ethnic minority)	-	-	-	-	-
Mental health diagnosis	0="yes", 1="no"	1 item: "Do you have any current psychiatric diagnosis?"	-	-	-	-	-
Perception of COVID risk							
COVID anxiety	Range: 0% - 100%	1 item: "How anxious are you about the coronavirus COVID-19 pandemic?"	-	-	-	-	-
Close people have been infected	0="no", 1="yes"	1 item: "Has someone close to you been infected by the coronavirus COVID-19?"	-	-	-	-	-
Perceived risk of infection	Mean-score, range: 0% - 100%	3 identical questions for different time-frames, e.g., "What do you think is your personal percentage risk of being infected with the COVID-19 virus over the following time periods? - In the next month"	0.93	0.92	0.97	0.95	0.94

Expected consequences of infection	Mean-score, range: 0 (not at all bad) – 100 (very bad)	Mean-score of 3 identical questions for different time-frames, e.g., "How bad do you think would be the consequences of you being infected with the COVID-19 virus over the following time periods? - In the next month"	0.96	0.96	0.97	0.97	0.96
Political mindedness							
Political orientation (higher values=more right-wing orientation)	1="Very left wing/ liberal" , 7= "Very right wing/conservative"	1 item: "Where do you feel your political views lie on a spectrum of left wing (or liberal) to right wing (or conservative)?"	-	-	-	-	-
Preferred sources of information (higher values=more social media)	1= "Always from major newspapers and/or TV channels", 5= "Always from social media"	1 item: "How do you find out about what is going on in the world?"	-	-	-	-	-
Specific Mistrust							
Pandemic persecutory threat (PPS)	factor-score, range 0-51.2	15 items, e.g., "I was sure someone wanted to infect me with COVID-19."	0.97	0.98	0.98	0.95	0.97
Pandemic paranoid conspiracy (PPS)	factor-score, range 0-20.1	6 items, e.g., "The government is lying to us about COVID-19."	0.93	0.94	0.94	0.93	0.92

Pandemic interpersonal mistrust (PPS)	factor-score, range 0-11.9	4 items, e.g., "I can't trust others to stick to the social distancing rules"	0.85	0.85	0.83	0.84	0.80
Pandemic paranoia global score (PPS)	factor-score, range 0-65.1	25 items (weighted sum of prior three PPS variables)	0.94	0.96	0.97	0.93	0.95
Vaccine conspiracy beliefs	Sum-score, range 7-49	7 items, e.g., "Vaccine safety data for COVID-19 is faked"	0.98	0.97	0.97	0.97	0.95
General mistrust							
Ideas of reference (RGPTS)	Sum-score, range 0-32	8 items, e.g., "I spent time thinking about friends gossiping about me"	0.95	0.94	0.92	0.94	0.91
Paranoid ideation (RGPTS)	Sum-score, range 0-40	5 items, e.g., "I was sure someone wanted to hurt me."	0.96	0.96	0.95	0.95	0.96
General conspiracy mentality (CMQ)	Sum-score, range 5-55	5 items, e.g., "there are secret organizations that greatly influence political decisions"	0.88	0.91	0.89	0.91	0.87
Social adversity							
Traumatic emotional neglect	0=no, 1=yes	1 item: "Did you ever experience any kind of emotional neglect?"	-	-	-	-	-

Traumatic psychological abuse	0=no, 1=yes	1 item: “Did you ever experience any kind of psychological abuse?”	-	-	-	-	-
Traumatic physical abuse	0=no, 1=yes	1 item: “Did you ever experience any kind of physical abuse?”	-	-	-	-	-
Traumatic sexual abuse	0=no, 1=yes	1 item: “Were you ever approached sexually against your will?”	-	-	-	-	-
Generalized beliefs							
Negative beliefs about self (BCSS)	Sum-score, range 0-24	6 items, e.g., “I am unloved”	0.84	0.86	0.84	0.86	0.87
Negative beliefs about others (BCSS)	Sum-score, range 0-24	6 items, e.g., “I am valuable”	0.90	0.91	0.90	0.87	0.91
Positive beliefs about self (BCSS)	Sum-score, range 0-24	6 items, e.g., “Other people are bad”	0.86	0.88	0.86	0.86	0.87
Positive beliefs about others (BCSS)	Sum-score, range 0-24	6 items, e.g., “Other people are trustworthy”	0.91	0.92	0.89	0.85	0.91
Perceived social rank (SCS)	Mean-score, range 1-10	11 semantic differentials, e.g., “Inferior:Superior”	0.94	0.95	0.95	0.94	0.94

Note: PPS = Pandemic Paranoia Scale; CMQ = Conspiracy Mentality Questionnaire; RGTPS = Revised Green Paranoid Thoughts Scale; Scale; BCSS = Brief Core Schema Scales; SCS = Social Comparison Scale.

Supplement 2. Correlation analysis by site

Table S2.

Point-biserial correlation between predictors and vaccine willingness in the total sample and within each site

	Total sample	UK	USA	Australia	Germany	HK	Total effect replicated in X subsamples
Socio-demographic data							
Age	**0.17*** **	**0.22*** **	**0.17*** **	0.08	**0.18*** **	**0.28*** **	4
Gender (0=male, 1=female)[a]	**-0.13*** **	**-0.12** **	**-0.15** **	**-0.10***	**-0.16*** **	**-0.12***	5
Size of current home city	**-0.07** **	**-0.13** **	**0.17*** **	0.09	0.04	0.02	1
Educational level (0="≥A-level", 1=primary/secondary)[b]	**-0.10*** **	-0.01	**-0.09***	-0.07	**-0.16*** **	**-0.12***	3
Annual income	**0.13*** **	0.08	**0.29*** **	**0.20*** **	**0.15** **	0.08	3
Employment status (0="working", 1="not working")	-0.04	-0.06	0.02	**-0.12***	-0.08	-0.09	-
Migrant status (0="no" vs. 1="yes")	0.02	-0.08	**0.09***	0.01	-0.04	0.04	-
Minority status (0="no" vs. 1="yes")	0.01	-0.06	0.04	-0.02	0.04	0.01	-

Number of minority group memberships	-0.02	**-0.12***	0.07	-0.04	0.01	-0.04	-
Mental health diagnosis (0="yes", 1="no")	0.01	0.01	0.02	0.11*	-0.02	-0.01	-
Perception of COVID risk							
COVID anxiety	**0.24*****	**0.15****	**0.36*****	**0.21*****	**0.35*****	0.03	4
Close people have been infected	**0.11*****	-0.02	**0.11***	0.09	**0.10***	-0.03	2
Perceived risk of infection	**0.19*****	**0.16*****	**0.23*****	**0.19*****	**0.28*****	-0.05	4
Expected consequences of infection	**0.15*****	**0.18*****	**0.24*****	**0.14****	**0.26*****	-0.09	4
Political mindedness							
Political orientation (higher values=more right-wing orientation)	**-0.10*****	0.01	**-0.21*****	**-0.20*****	**-0.14****	**0.25*****	3
Preferred sources of information (higher values=more social media)	**-0.14*****	**-0.24*****	**-0.15****	0.02	**-0.19*****	**-0.21*****	4
Specific Mistrust							
Pandemic persecutory threat (PPS)	**0.06****	-0.03	**0.12***	**0.20*****	-0.06	0.04	2
Pandemic paranoid conspiracy (PPS)	**-0.39*****	**-0.53*****	**-0.34*****	**-0.21*****	**-0.49*****	**-0.38*****	5
Pandemic interpersonal mistrust (PPS)	**0.11****	0.05	**0.22*****	**0.13****	0.09	-0.08	2

Pandemic paranoia global score (PPS)	**-0.05***	**-0.18*****	0.03	**0.11***	**-0.19*****	-0.10	2
Vaccine conspiracy beliefs	**-0.56*****	**-0.68*****	**-0.57*****	**-0.49*****	**-0.64*****	**-0.41*****	5
General mistrust							
Ideas of reference (RGPTS)	**-0.05***	**-0.16*****	0.03	0.05	-0.08	-0.02	1
Paranoid ideation (RGPTS)	**-0.04**	**-0.17*****	0.04	0.00	**-0.10***	0.02	2
General conspiracy mentality (CMQ)	**-0.35*****	**-0.34*****	**-0.34*****	**-0.27*****	**-0.47*****	**-0.36*****	5
Social adversity							
Traumatic emotional neglect	**0.08*****	**0.10***	**0.11***	0.05	0.08	-0.02	2
Traumatic psychological abuse	**0.12*****	**0.18*****	**0.11***	**0.11***	**0.13****	0.08	4
Traumatic physical abuse	**0.05***	0.01	0.08	**0.10***	**0.11***	-0.05	2
Traumatic sexual abuse	**0.08*****	**0.12***	**0.10***	**0.10***	0.07	0.08	3
Generalized beliefs							
Negative beliefs about self (BCSS)	**-0.10*****	**-0.15*****	**-0.13****	**-0.11***	-0.05	-0.04	3
Negative beliefs about others (BCSS)	**0.07*****	**0.12***	**0.14****	**0.12***	0.05	**0.20*****	3
Positive beliefs about self (BCSS)	**-0.15*****	**-0.22*****	**-0.23*****	**-0.24*****	**-0.14****	0.00	4
Positive beliefs about others (BCSS)	**0.18*****	**0.17*****	**0.24*****	**0.19*****	**0.13****	**0.19****	5

Perceived social rank (SCS)	**0.11*** **	**0.12***	**0.17*** **	**0.20*** **	0.07	**0.21*** **	4

Note: a) to avoid bias due to low cell counts the variables sex and gender were combined into a dichotomized variable to reflect the gender a participants most likely reads as at present, leading to a recoding for 16 participants (0.63%); b) educational level was dichotomized with GCSE or lower categorized as low educational level and everything else as high educational level; PPS = Pandemic Paranoia Scale; CMQ = Conspiracy Mentality Questionnaire; RGTPS = Revised Green Paranoid Thoughts Scale; Scale; BCSS = Brief Core Schema Scales; SCS = Social Comparison Scale. Significant results are printed in bold. Correlations printed in red show significant results with an opposite correlation when compared to the total sample.

Supplement 3. Hyperparameter tuning of the machine learning models

Strategy for hyperparameter tuning

The hyperparameter tuning was conducted in a nested-cross-validation procedure with eight outer and four inner folds to identify the best hyperparameter configuration for each model. Due to the fact that classes were skewed we used random undersampling to prevent models potentially optimizing for the majority class. For the hyperparameter tuning we balanced classes on the whole dataset before starting the nested-cross-validation.

In nested cross-validation outer folds are split in training and testing folds. We split the training data of each outer fold in four inner folds consisting of a training and validation data set each. Hyperparameter configurations were cross validated in the inner folds and the best configuration in the inner fold was applied to the outer fold testing data. We used a randomized search over our hyperparameter space and optimized for the metric accuracy. Random forest classifiers could vary in their number of decision trees (50, 100, 200, 500, 1000, 1500), maximum depth of decision trees (5, 10, 15, 20, 25, 30, 35, 40, 45, 50, 55, 60, 65, 70, 75, 80, 85, 90, 95, 100, No restriction), maximum features allowed to use per split (20%, 40%, 60%, 80%), and the minimum samples required at each leaf node (10, 15, 20, 25, 30). Furthermore, we always used bootstrapping but varied the bootstrap sample size drawn from each training dataset (25%, 50%, 75%, 100%). Additionally, we pruned decision trees after calculation by varying the degree of model complexity penalization with the use of cost-complexity pruning (0.005, 0.01, 0.0125, 0.015, 0.0175). Our hyperparameter space resulted in 50400 possible configurations and we randomly searched 20% of this hyperparameter space (10080 configurations). Results of the hyperparameter tuning for all models are summarized in Table S2.

If multiple test folds yielded equal test accuracy we always chose the hyperparameter configuration with the least number of decision trees to optimize for speed of prediction as well. Following hyperparameter tuning, the leave-one-site-out and leave-one-person-out cross-

validation of the models with their respective best hyperparameter configuration was performed using random undersampling on each training fold before model calculation, whereas the test folds were based on the full data set.

Table S3

Hyperparameter tuning results and final hyperparameter configuration for each model

Variables	Mean test accuracy	Mean training accuracy	Number of trees	Max. depth	Max. features per split	Min. samples per leaf	bootstrap	Ccp alpha
Standard model (without middle category)								
All variables included	82.35%	85.74%	200	80	80%	10	100%	0.005
Vaccination conspiracy belief excluded from model	75.81%	82.22%	100	30	80%	10	25%	0.005
Specific/General mistrust excluded from model	68.62%	75.85%	1000	55	20%	10	100%	0.005
Prediction using the most relevant variables								
12 best variables	82.27%	85.41%	1500	35	80%	10	100%	0.005
7 best variables	82.72%	84.95%	500	100	20%	10	75%	0.005
Model with middle category to not willing								
All variables included	77.67%	80.26%	500	10	80%	10	50%	0.005
Model with middle category as separate class (Multi-label random forest)								
All variables included	59.05%	70.21%	200	70	40%	10	50%	0.005

Note. Ccp alpha = alpha value for cost-complexity pruning

Supplement 4. Strategy for variable importance analyses

We decided to use permutation feature importance because impurity-based feature importance measures have been shown to be biased[47] and permutation feature importance allowed us to investigate variable importance on the test dataset instead of on the training dataset. We used permutation feature importance for the site cross-validation analysis. Each variable of the testing data was permuted ten times. The accuracy of models was then re-evaluated with the permutation of each variable and the overall accuracy of each resulting model with permutation was juxtaposed with the original model. For each variable, a score of the mean of the 10 differences in accuracy between the original model and the model with one of the 10 permutations of this variable was calculated to estimate its importance in a given model in terms of reduction in model-accuracy without meaningful information from this variable.

To avoid any bias by relying solely on permutation feature importance for variable selection we additionally performed the calculation of SHapley Additive exPlanations (SHAP[45]) to compare whether most important variables mirrored the results of permutation feature importance. SHAP is an additive feature attribution method which has its theoretical basis in game theory and provides a model-agnostic approach to explain the output of machine learning models. Similar to our permutation feature importance analysis we calculated SHAP for each individual in the test folds of the leave-one-site-out cross validation analysis. For our analysis we used the treeSHAP[48] algorithm because it delivers fast and exact estimations of SHAP values for tree ensemble methods like random forests. To obtain a measure of global variable importance we then averaged the absolute SHAP values over all individuals to obtain a mean absolute SHAP value for each variable.

Supplement 5 – descriptive values of all predictors

Table S4

Mean values and standard deviations for all psychosocial predictors by site

Variables	UK (n=512)		USA (n=535)		AU (n=502)		GE (n=516)		HK (n=445)	
	M/%	SD	M/%	SD	M/%	SD	M/%	SD	M/%	SD
Perception of COVID risk										
COVID anxiety	57.00	28.08	53.63	32.80	51.93	29.46	58.66	29.56	55.98	24.29
Close people have been infected	40.63%	-	41.50%	-	13.94%	-	28.29%	-	6.74%	-
Perceived risk of infection	4.82	2.25	4.02	2.55	4.03	2.79	4.76	2.50	4.06	2.25
Expected consequences of infection	42.75	26.55	37.48	30.70	48.75	32.14	48.20	30.24	46.54	27.44
Political mindedness										
Political orientation (higher values= more right-wing orientation)	3.66	1.43	4.17	1.72	3.57	1.36	3.65	1.17	3.74	1.18
Primary source of information (higher values=social media)	2.41	1.24	2.46	1.31	2.58	1.28	2.60	1.19	2.37	1.29
Specific Mistrust										
Pandemic persecutory threat (PPS)	2.48	7.03	4.70	10.71	7.20	12.20	3.11	7.11	5.27	9.05
Pandemic paranoid conspiracy (PPS)	3.85	3.22	3.48	3.44	4.10	3.25	2.93	3.01	3.52	2.73
Pandemic interpersonal mistrust (PPS)	3.66	4.83	4.71	5.92	5.50	5.94	4.05	5.20	6.01	5.36

General pandemic paranoia (PPS)	6.68	9.05	9.09	12.97	12.18	14.60	6.94	9.07	10.42	11.15
Vaccine conspiracy beliefs	16.32	11.39	20.55	12.77	22.41	12.31	18.62	11.83	19.91	11.30
General mistrust										
Ideas of reference (RGPTS)	6.46	8.59	6.77	8.69	11.73	8.52	7.13	8.60	9.17	7.07
Paranoid ideation (RGPTS)	6.03	9.66	6.85	10.42	12.01	11.02	5.72	9.11	8.52	9.67
General conspiracy mentality (CMQ)	29.95	10.43	31.30	12.43	34.09	10.35	24.45	12.44	34.61	9.73
Social adversity										
Traumatic emotional neglect	30.66%	-	33.64%	-	61.75%	-	44.19%	-	45.39%	-
Traumatic psychological abuse	25.59%	-	34.95%	-	50.80%	-	36.82%	-	30.34%	-
Traumatic physical abuse	20.90%	-	30.52%	-	39.64%	-	30.62%	-	12.41%	-
Traumatic sexual abuse	18.16%	-	28.41%	-	38.84%	-	19.96%	-	17.08%	-
Generalized beliefs										
Negative beliefs about self (BCSS)	2.92	4.70	2.60	4.73	5.35	5.81	3.29	4.85	3.48	5.31
Negative beliefs about others (BCSS)	4.52	5.83	5.76	6.79	6.63	6.97	5.55	5.96	2.88	5.17
Positive beliefs about self (BCSS)	9.83	6.15	14.10	6.94	8.72	6.39	12.80	6.46	11.38	6.21
Positive beliefs about others (BCSS)	9.83	6.09	11.14	6.76	8.81	6.39	10.81	5.87	8.54	6.02
Perceived social rank (SCS)	5.54	1.71	6.49	1.99	5.17	2.00	6.34	1.80	6.08	1.50

Note: PPS = Pandemic Paranoia Scale; CMQ = Conspiracy Mentality Questionnaire; RGTPS = Revised Green Paranoid Thoughts Scale; Scale; BCSS = Brief Core Schema Scales; SCS = Social Comparison Scale.

Supplement 6 – Machine Learning models predicting vaccine willingness based on the full dataset including the mid-category of indecisive participants

Table S5

Cross-validation results for the machine learning model based on all variables in the full sample with indecisive participants labelled as vaccination hesitant

Cross validation	Sensitivity	PPV	Specificity	NPV	BAC	TAC
Leave-one-site-out cross validation	0.77	0.77	0.68	0.69	0.73	0.74
Leave-one-person-out cross validation	0.77	0.81	0.75	0.71	0.76	0.77

Note. PPV = positive predictive value, i.e. the frequency true positive tests among all positive tests; NPV = negative predictive value, i.e. the frequency true negative tests among all negative tests; BAC = balanced accuracy, i.e. the average of sensitivity and specificity; TAC = total unweighted.

Table S6

Cross-validation results for multi-label random forest classifiers predicting answers for the leave-one-site-out and the leave-one-person-out cross-validation

	Cross validation on left-out site				Cross validation of left-out person			
Classification Category	Recall	Precision	BAC	TAC	Recall	Precision	BAC	TAC
Refusing to take a vaccine (1-2)	0.65	0.63	0.55	0.61	0.65	0.64	0.59	0.64
Indecisive (3)	0.34	0.22			0.42	0.26		
Willing to take a vaccine (4-5)	0.67	0.79			0.69	0.84		

Note. Recall = The frequency of correctly classified persons among all persons in the dataset with this class (similar to sensitivity); Precision = The frequency of correctly classified persons among all persons predicted as this class (similar to positive predictive value); BAC = balanced accuracy, i.e. the average of all recall values; TAC = total unweighted accuracy.
